# Conformational Alterations of the Cell Surface of Monomeric and Dimeric β2m-Free HLA-I (Proto-HLA) May Enable Novel Immune Functions in Health and Disease

**DOI:** 10.3390/cimb46070416

**Published:** 2024-07-04

**Authors:** Mepur H. Ravindranath, Narendranath M. Ravindranath, Carly J. Amato-Menker, Fatiha El Hilali, Edward J. Filippone

**Affiliations:** 1 Department of Hematology and Oncology, Children’s Hospital, Los Angeles, CA 90027, USA; 2 Terasaki Foundation Laboratory, Santa Monica, CA 90064, USA; 3 Norris Dental Science Center, Herman Ostrow School of Dentistry, University of Southern California, Los Angeles, CA 90089, USA; nravindr@usc.edu; 4 Department of Microbiology, Immunology and Cell Biology, School of Medicine, West Virginia University, Morgantown, WV 26506, USA; carly.j.amato@gmail.com; 5 Medico-Surgical, Biomedicine and Infectiology Research Laboratory, The Faculty of Medicine and Pharmacy of Laayoune & Agadir, Ibnou Zohr University, Agadir 80000, Morocco; f.elhilali@uiz.ac.ma; 6 Division of Nephrology, Department of Medicine, Sidney Kimmel Medical College, Thomas Jefferson University, Philadelphia, PA 19145, USA; kidneys@comcast.net

**Keywords:** human leukocyte antigens, beta2-microglobulin, heavy chains, monomers, homodimers, heterodimers, proto-HLA, killer immunoglobulin-like receptors, leukocyte immunoglobulin-like receptors

## Abstract

Human leukocyte antigens (HLAs) are polymorphic glycoproteins expressed on the cell surface of nucleated cells and consist of two classes, HLA class I and HLA class II. In contrast, in mice, these molecules, known as H-2, are expressed on both nucleated cells and erythrocytes. HLA-I molecules (Face-1) are heterodimers consisting of a polypeptide heavy chain (HC) and a light chain, B2-microglobulin (B2m). The heterodimers bind to antigenic peptides and present them to the T-cell receptors of CD8+ cytotoxic T lymphocytes. The HCs can also independently emerge on the cell surface as B2m-free HC monomers without peptides (Face-2). Early investigators suggested that the occurrence of B2m-free HCs on the cell surface resulted from the dissociation of B2m from Face-1. However, others documented the independent emergence of B2m-free HCs (Face-2) from the endoplasmic reticulum (ER) to the cell surface. The clustering of such HC molecules on either the cell surface or on exosomes resulted in the dimerization of B2m-free HCs to form homodimers (if the same allele, designated as Face-3) or heterodimers (if different alleles, designated as Face-4). Face-2 occurs at low levels on the cell surface of several normal cells but is upregulated on immune cells upon activation by proinflammatory cytokines and other agents such as anti-CD3 antibodies, phytohemagglutinin, and phorbol myristate acetate. Their density on the cell surface remains high as long as the cells remain activated. After activation-induced upregulation, Face-2 molecules undergo homo- and heterodimerization (Face-3 and Face-4). Observations made on the structural patterns of HCs and their dimerization in sharks, fishes, and tetrapod species suggest that the formation of B2m-free HC monomers and dimers is a recapitalization of a phylogenetically conserved event, befitting the term Proto-HLA for the B2m-free HCs. Spontaneous arthritis occurs in HLA-B27+ mice lacking B2m (HLA-B27+ B2m−/−) but not in HLA-B27+ B2m+/+ mice. Anti-HC-specific monoclonal antibodies (mAbs) delay disease development. Some HLA-I polyreactive mAbs (MEM series) used for immunostaining confirm the existence of B2m-free variants in several cancer cells. The conformational alterations that occur in the B2m-free HCs enable them to interact with several inhibitory and activating receptors of cellular components of the innate (natural killer (NK) cells) and adaptive (T and B cells) immune systems. The NK cells express killer immunoglobulin-like receptors (KIRs), whereas leukocytes (T and B lymphocytes, monocytes/macrophages, and dendritic cells) express leukocyte immunoglobulin-like receptors (LILRs). The KIRs and LILRs include activating and inhibitory members within their respective groups. This review focuses on the interaction of KIRs and LILRs with B2m-free HC monomers and dimers in patients with spondylarthritis. Several investigations reveal that the conformational alterations occurring in the alpha-1 and alpha-2 domains of B2m-free HCs may facilitate immunomodulation by their interaction with KIR and LILR receptors. This opens new avenues to immunotherapy of autoimmune diseases and even human cancers that express B2m-free HCs.

## 1. Introduction

Human leukocyte antigens (HLAs) and murine histocompatibility antigens (murine H antigens) are the major histocompatibility complex (MHC) antigens. HLA antigens are further distinguished in humans as HLA class I (HLA-I) and HLA class II (HLA-II). HLA-I is expressed on the cell surface of nucleated cells, leukocytes, fibroblasts, and platelets but not on erythrocytes. In contrast, mouse MHC class I antigens, henceforth referred to as H-2, are found on both leukocytes and erythrocytes.

HLA-I molecules, highly polymorphic cell surface glycoproteins, are described as heterodimers, consisting of two polypeptides, a heavy chain (HC), and a noncovalently associated beta 2-microglobulin (B2m) [1,2], whereas HLA-II molecules are homodimers of two HCs. The HLA-I isoforms on the cell surface consist of a polymorphic 41 kDa HC. They are embedded in the membrane via a hydrophobic stretch of amino acids near its carboxy terminus [2]. The extracellular domain of the isoforms consists of B2m, a non-polymorphic, non-glycosylated light chain of about 12 kDa [1]. The HC has three external domains (α1, α2, and α3), each with about 90 amino acids, a short α3-transmembrane domain, and a cytoplasmic tail peptide of about 35 amino acids. Crystallographic and biochemical analyses indicate that B2m is associated with the α3 external domain. The glycosylated domain of the cell surface HC consists of a single asparagine-linked oligosaccharide composed of N-acetyl-glucosamine, mannose, galactose, fucose, and sialic acid [3]. The natural immunologic function of HLA-I is to bind antigenic peptides together with B2m for presentation to the T-cell receptor (TCR) of CD8+ cytotoxic T lymphocytes, whereas that of HLA-II is to present antigen peptides to the TCR of CD4+ helper T cells. 

The HLA gene complex (more than 220 genes) is comprised within the 6p21.3 region of the short arm of human chromosome 6. HLA-I comprises distinct loci, each of which can be present as many different alleles. The number of discovered alleles increases every year. As of September 2023, the number of alleles of different HLA-I loci identified was as follows: HLA-A (8012), HLA-B (9573), HLA-C (7995), HLA-E (350), HLA-F (81), and HLA-G (151). 

Our focus in this review of literature is to document the existence of two forms of B2m-free HCs of HLA-I isoforms. To distinguish the different forms of B2m-free HCs, the term Face-1 is coined for the intact B2m-associated HCs of HLA-I molecules, and the term Face-2 is given for B2m-free HLA HCs [4]. Further critical examination of the literature revealed that Face-2 or B2m-free HC can dimerize with its allelic Face-2 as a homodimer (Face-3), or dimerize with a B2m-free HC of another allele as a heterodimer (Face-4). Most importantly, this review focuses on the evidence that supports the contention that the monomeric B2m-free HC can arise de novo, particularly after inflammation, autoimmune diseases, and particularly in human cancers. 

Briefly reviewing the literature on HLA-I isoforms in bony fishes, sharks, amphibians, reptiles, birds, and mammals and during vertebrate evolution (see reference 1, 194–206 in [4]) and based on the findings that inflammation, autoimmune diseases, and human cancers promote de novo expression of Face-2 and trigger further the formation of noncovalent transient HC-dimers (Face-3 and Face-4), we coined the term “Proto-HLA” for these different forms of B2m-free HCs (Face-2–4). Based on similarities in the amino acid sequences between Proto-HLA and the HCs of HLA-I and HLA-II, we suggested that they could be the evolutionary progenitors of both the classes of HLAs [4]. This review further elucidates that the cell surface monomeric B2m-free HCs (the Proto-HLAs) can perform novel immune functions, differently from that of heterodimeric HLA-I isoforms.

## 2. Human and Murine Cell Surfaces May also Express HLA-I HCs without B2m

In general, the structure of HLA-I was considered to be simply a heterodimer until 1979. However, several investigators [5,6,7,8,9,10,11] have shown that the human Daudi and murine R1E and EL4 cell lines do not express B2m-associated HLA-I molecules at the cell surface. Similarly, B2m-deficient NK cells are found in H-2K^b^-deficient and H-2D^b^-deficient mice [12]. Parnes and Seidman [8] clarified that these human and murine cell lines have mutated B2m genes, thereby failing to synthesize B2m and expressing intact HLA-I on the cell surface. When the B2m protein was introduced into these cell lines by fusion with another cell, it restored the cell surface expression of intact HLA-I molecules. An anti-H serum, generated against the papain-solubilized HLA-B HC, has been used to document the HCs in B2m-deficient Daudi cell lines. Notably, the HCs remained intracellular at all times [9]. 

In the murine system, an exception to this tenet was noted. Potter et al. [10,11] found that the HCs of the mouse MHC class I antigen H-2D^b^ was expressed at the cell surface of the EL4/Mar line but were not associated with B2m, although B2m was identified within the cell. While experimenting further on the requirement of B2m for the expression of the cell surface H-2D^b^, Allen et al. [7] concluded that the D^b^ heavy chain does not require B2m for transport to the cell surface. Furthermore, they further documented that both D^b^-specific cytotoxic T cells and most of the D^b^-specific monoclonal antibodies fail to recognize the HCs on the cell surface; they concluded that the Db heavy chain does not retain the native conformation in the absence of B2m. Indeed, this is the first report documenting conformational variation in the B2m-free HCs, and this finding is supported by several others, who claim that B2m-free HCs bind to exogenous B2m poorly [13,14], possibly because such B2m-free HCs assume a different conformation than those associated with B2m [15,16].

## 3. Early Documentation of HLA-I B2m-Free HC Monomers on the Human Cell Surface

In 1979, Krangel, Orr, and Strominger [17] analyzed the assembly and maturation of HLA-A and HLA-B antigens in the human B lymphoblastoid cell line T5-1 with the murine monoclonal antibody (mAb) W6/32, which recognizes a determinant present on all HLA HCs when they are associated with B2m [18,19]. In addition, they also used an anti-H rabbit serum developed against B2m-free HCs and another anti-rabbit serum raised against human urinary B2m. They reported that the anti-H-reactive heavy chains are detectable on the surface of T5-1. They speculate that the B2m-free HCs may be present due to the movement of the HCs from inside the cell to the cell surface or would have resulted by the dissociation of W6/32-reactive B2m-associated HCs once they reach the cell surface. This is the first direct report on the occurrence of HC monomers on the cell surface of a human cell line.

Another interesting report on the cell surface expression of B2m-free HLA HCs emerged from the works of Bushkin et al. [20,21], carrying out biochemical characterization of the α/β T cell receptor molecules expressed on leukemia cells in patients with Sezary syndrome (SU). Human SU leukemia cells express cell surface α/β T cell antigen receptor molecules (chains) with 38 and 43 kDa molecular masses. They also studied the cell surface expression of HLA-I molecules employing mAb W6/32, which specifically recognized B2m-associated HLA HCs. In addition, they also used another mAb (A1.4) developed in their laboratory using Con-A activated T cells as the immunogen. The mAb A1.4 strikingly differs from that of W6/32 in that it recognizes B2m-free HCs. The A1.4 mAb recognized B2m-free HLA HCs precipitated from the “W6/32-precleared SU leukemia cell lysate”. In addition to the 43 kDa molecule, the HCs are found to be noncovalently associated with another 38 kDa molecule in the form of a dimer. Using the mAb A1.4, Bushkin et al. [22] demonstrated that the CD8 molecules are also associated with B2m-free HCs on the cell surface of human T cells activated by Con A or allogeneic stimulation, but not on untreated or unactivated T cells. 

## 4. Does the Cell Surface of B2m-Free HLA HCs Represent a Separate Entity (as Proto-HLA) of the HLA Class? 

Using mAb LA45, Schanbl et al. [23] have immunoprecipitated the LA45 antigen from activated T lymphocytes, studied its protein sequence, and compared it with the HLA HC data banks to reveal that the antigen is indeed an HC of an HLA-I allele. The mAb LA45 recognized the shared sequence of several HCs of HLA-I alleles. Using a monoclonal antibody (mAb W6/32) that recognizes and immunoprecipitates the B2m-associated HCs of HLA. Under reducing conditions, a band of 45 kDa B2m and a protein of 12 kDa were formed. Interestingly, mAb LA45 reacted with the 45 kDa molecule but did not recognize B2m (12 kDa), confirming that mAb LA45 is specific for B2m-free HLA HCs. Interestingly, LA45 antigens are virtually absent from the surface and cytoplasm of resting peripheral blood and bone marrow mononuclear and polymorphonuclear cells. However, after stimulation with PHA, the expression of the LA45 antigen was induced on T cells within 24 h. Furthermore, they showed that cap formation induced by W6/32 or anti-B2m antibodies did not cap LA45 molecules, and the LA45 antibody did not induce capping of HLA class I molecules. Evidently, LA45 is a non-B2m-associated HLA class I alpha chain and is structurally different from conventional heteromeric HLA molecules recognized by W6/32 and anti-B2m. Furthermore, these observations confirm the previous speculation that activated T cells express structurally altered non-B2m-associated HCs. Whether these structures represent a separate alloantigen system remains to be seen. In addition, they report that the mitogen-stimulated (mononuclear cells) of 12 normal donors showed LA45 reactivity, although they had different HLA phenotypes. In conclusion, it is clarified that on activated T cells and possibly other cells, HLA class I can be expressed in association with B2m as well as in a free, conformationally altered form. This investigation is another direct report to document that B2m-free HLA HCs can be expressed on the human cell surface in a conformationally altered form.

Strikingly, a similar inference was derived by Demaria et al. [24]. They studied the kinetics of B2m-free HC expression on human peripheral blood T cells after stimulating with phorbol myristate acetate (PMA) and detected B2m-free HCs after 8 h. Notably, B2m-free HCs are also expressed on the T cells activated with anti-CD3 mAb and phytohemagglutinin (PHA). The expression levels of B2m-free HCs correlated with the activation levels as determined by the expression of the IL-2 receptor (R). On PMA-activated resting T cells, they used brefeldin A (BFA) to block the expression of IL-2R, while B2m-associated HLA HCs were monitored with mAb W6/32 and B2m-free HCs were monitored with mAb HC10. They suggest that the newly expressed cell surface B2m-free HCs originated from surface B2m-associated HCs based on the following findings: First, BFA blocked the expression of B2m-free class I heavy chains induced on T cells by PMA if added within 4 h after the delivery of activation signals. In association with this, BFA prevented the PMA-induced upregulated expression of B2m-associated MHC class I molecules. Second, BFA failed to prevent the reappearance of B2m-free class I heavy chains on activated T cells following their selective removal with trypsin. Demaria and Buskin [25] postulate that the acidic environment of endosomes may favor the dissociation of B2m and HLA-I HCs and, as a consequence, B2m-free HCs are recycled to the cell surface. 

Demaria et al. [24] used mAbs W6/32 and HC10 to document B2m-associated HLA and B2m-free HC monomers and dimers, respectively. They conclude that B2m-free HCs observed on the cell surface may result from the dissociation of B2m-associated HCs. However, Tran et al. have shown in Table 4 of their publication [26] to clarify the following facts:

- HLA molecules recognized by W6/32 as free α chains (even after boiling in SDS) are as follows: B7, B8, B13, B15, B18, B22, B35, B38, B39, B45, B50, B51, B55, B57, B60, B62, B63.
- HLA molecules recognized by W6/32 only as SDS-resistant noncovalent complexes with β2m (but not as free HCs) are as follows: B27, B*2701, B*2702, B*2705, B49.

Based on these and other findings, they conclude that W6/32, though considered to be a monomorphic, pan-HLA-A, -B, -C mAb reactive to a conformational epitope present in intact complexes of α chains with B2m and peptides, does recognize an epitope preserved in free nonreduced α chains of most HLA-B antigens. In light of these findings, it is reasonable to challenge the previous notion that the emergence of B2m-free HCs results from dissociation of the HCs from B2m-associated intact HLA. Indeed, many subsequent reports (vide infra) have revealed that B2m-free HCs are generated on the cell surface without dissociating B2m from intact HLA-I.

## 5. Expression of Cell Surface B2m-Free HLA-B27 HCs in Transgenic Mice 

The cell surface expression of B2m-free HC of HLA-I is further clarified by studies on the disease associations of HLA-B27. Possession of HLA-B27 is strongly associated with the development of the spondyloarthritides and a group of related diseases, including idiopathic ankylosing spondylitis (AS), Reiter’s disease, spondylitis accompanying chronic inflammatory bowel disease, psoriatic arthritis, and juvenile chronic polyarthritis [27,28]. Khare, Luthra, and David [29] documented that a pathogenic role of HLA-B27 HCs in murine models of spondylarthritis occurred independent of B2m by introducing the HLA-B27 HC transgene into the B2m-deficient mice to determine whether processing, assembly, transport, and cell surface expression of the HLA-B27 HC may play a role in the disease process. To determine whether the HLA-B27 molecule may reach the cell surface in B27+ B2m−/− mice, splenocytes were stimulated in vitro, and the expression of free HCs of B27 was determined. They reported (with Figure 6A,B [29]) the following findings:

(1) A low-level expression of the HCs of HLA-B27 was detected on the cell surface of Con A-stimulated splenocytes.
(2) The presence of HLA-B27 HCs on Con A-stimulated splenocytes was further confirmed by observing >50% of lysis of B27+ B2m−/− targets with anti-HLA-B27 CTLs in a ^51^Cr release assay.

The presence of HCs on the cell surface after stimulation further suggested that environmental antigens can stimulate cells to express (B2m)-free HCs on the cell surface. The authors pointed out the occurrence of the disease in B27+ B2m−/− mice due to the aberrant expression of autoimmune reactivity of the HCs.

The concept of arthritis development in mice expressing B2m-free HCs of HLA-B27 is further supported in the experiments of Khare et al. [30], for which they employed mAb HC10 specific for B2m-free HCs [31]. Indeed, the amino acid sequence 55 to 64 of the B2m-free HCs of classical HLA-Ia alleles is the specific epitope of the mAb HC10 [32]. The incidence of spontaneous development of arthritis decreased by 33% in HC10-treated animals compared with mAb ME1 (specific for B2m-associated HLA)-treated and PBS-treated animals [30]. These observations led to the postulate that at disease onset, the pathological effects or severity of B2m-free HCs or Proto-HLA, as they occur in autoimmune diseases and human cancers, can be minimized or eliminated by administration of HC-specific mAbs such as HC10.

Another mAb (HD6), that exhibit the same affinity as HC10, is specific for monomeric and dimeric variants of B2m-free HC of HLA-B27, was generated using recombinant B2m-free B27 heavy-chain dimers (B27_2_) [33]. The mAb HD6 binds to recombinant HLA-B27_2,_ HLA-B27 dimers and monomeric heavy chains. These molecules were B2m-free because they were not reactive to B2m-specific mAb BBM.1. The mAb HD6-stained monocytes, peripheral blood mononuclear cells, and synovial fluid mononuclear cells from spondyloarthritides (SpA) patients. The monocytes from SpA patients exhibited significantly more HD6 positivity than did those from B27-negative or B27-positive controls. Variable staining of patient B cells was observed. Interestingly, McHugh et al. [34] observed that HD6 and HC10 reactivity levels increased on monocytes concomitant with disease progression in transgenic rats. All these findings conclusively prove the existence of B2m-free Proto-HLA of B27 in human cells.

## 6. Expression of Cell Surface B2m-Free HCs of Other Alleles of HLA-I in Humans

Immunostaining peripheral blood cells and synovial cells from 38 Chinese AS patients and 10 RA patients with mAb HC10, showed significant increases in B2m-free HCs in monocytes and in 90% of the synovial cells of patients, compared to those of healthy controls [35]. Interestingly, after culturing monocytes isolated from healthy controls (all B27 negative) for a week with LPS, IL-4, GM-CSF, or TNF-α, HC10 reactivity was dramatically increased, suggesting that different proinflammatory cytokines can augment the expression of B2m-free monomers (Proto-HLAs). The matured monocytes expressed variable levels of B2m-free monomers of different alleles of HLA-I. Similarly, Lan et al. [36] reported that circulating monocytes showed higher expressions of HC10 compared with circulating lymphocytes (*p* < 0.05). Psoriatic patients with arthropathy showed elevated expression of HC10 on peripheral blood monocytes compared with those without arthropathy (*p* < 0.05). Among the arthropathic group, those without the human leucocyte antigen (HLA)-B27 allele showed even higher expression of HC10-positivity on circulating monocytes compared with those possessing HLA-B27 (*p* < 0.05). 

Raine et al. [37] also documented B2m-free HC levels by immunostaining the peripheral blood lymphocyte subpopulations and extravillous trophoblasts. They had higher levels of B2m-free HC expression in reactive arthritis patients than in healthy controls and RA patients. Notably, the B2m-free HC reactivity is not restricted to HLA-B27. LPS-treated leukocytes from arthritic patients had a significant upregulation of B2m-free HCs compared with an HLA-B27+ control population.

During inflammation in SpA, increased expression of B27 was observed. Comparing B27 B2m-free HCs, expressed as both monomers and dimers, in the human KG-1 cell line (expressing HLA-A30, -A31, B35, Cw-4 with transfected B*27:05), B27 transgenic rat antigen-presenting cells (APCs) and HLA-B27- and non-HLA-B27-expressing monocyte and dendritic cell (DC) populations from four different individuals, the formation of HLA-B27 heavy-chain dimers were documented in the KG-1 cell line and in HLA-B27-positive monocyte-derived human dendritic cells [38]. Interestingly, very low levels of dimeric bands are observed in the HLA-B27-negative dendritic cells, suggesting HC dimerization, may be relevant to other alleles. 

Boyson et al. [39] indicated that cell surface HLA-G, which “arrives at the cell surface as a monomer”, dimerizes with other HLA-I monomers. They further showed that HLA-G dimerization might depend on a high cell-surface density of HLA-G because dimers were detected on high-expressing HLA-G transfectants but not on the low-expressing JEG-3 choriocarcinoma cell line. Thus, conditions favoring high local concentrations of class I MHC proteins, such as lipid raft formation or clustering with a ligand at a cell–cell synapse, may facilitate HLA-G dimerization. Their observations suggest that most of the HLA-G monomers are on the cell surface, and the dimerization may occur as a two-step process. First, a stable, noncovalent dimer is formed, following which Cys-42-mediated disulfide bond formation occurs. 

Seitz et al. [40] used mAb HCA2, generated with B2m-free HC, that reacts specifically with B2m-free HCs and not with B2m-associated HCs. HCA2 recognizes an epitope encompassing positions 76–83 in HLA-E and -F as well as select HLA-A, -B, and -C HCs, and these sequences are masked by B2m in intact B2m-associated HLA molecules [41]. Using the peptide sequence for inhibition of mAb binding, they documented the cell surface expression of B2m-free HCs, not only of B27, but also HCs of HLA-B73, HLA-B7, HLA-C, HLA-E, and HLA-F. Similarly, based on immunoprecipitation experiments performed with a panel of HLA-F monoclonal antibodies, namely 3D11, 4B4, and 4A11, Goodridge et al. [42] also concluded that more than one form of HLA-F is expressed on the cell surface. Indeed, the in vitro studies carried out by Dirscherl et al. [43] with isolated HCs in conjunction with molecular docking and dynamic simulations suggested that the α3 domain of one monomer can dimerize with another monomer.

## 7. B2m-Free Monomeric HCs (Face-2) Expressed on Cancer Cell Surfaces

Giacomini et al. [44] studied the IFN-γ induced (at 10, 100, and 100 U/mL) expression of B2m-associated HLA HCs (Face-1) and B2m-free HLA HCs (Face-2) on the cultured human melanoma cell line Colo 38, using mAbs NAMB-1 (B2m-specific), W6/32 (B2m-associated HLA binding), and mAb Q1/28 (which recognizes a monomorphic determinant expressed on the B2m-associated and B2m-free HCs). After 24 h of IFN-γ-incubation, the percent increase in reactivity was more marked with the mAb Q1/28 than with the other two mAbs. No marked differences were observed among the three doses of IFN-γ. Prolongation of the incubation to 48 h increased the extent of binding of mAb Q1/28. They proposed that IFN-γ may render the cells permissive to monomeric assembly of HCs of HLA. Furthermore, mAb L31 (specific for B2m-free HCs) was used to identify a low level of cell surface expression of Face-2 (B2m-free HCs) of HLA-C on normal epidermis, breast, lung, bronchi, esophagus, stomach, ilium, colorectum, gall bladder, urinary bladder, seminal vesicles, ovarian epithelia, endometrium, and thymus tissues. L31-positivity was also observed on carcinomas of the epidermis, urinary bladder, thymus, and kidney; adenocarcinoma of the breast, thyroid, stomach, colorectum, liver, pancreas, ovary, endometrium, and prostate; and mixed histotypes of the brain and bronchi.

Similarly, L31 was used in flow cytometry to document B2m-free HC on the cell surface of the neuroblastoma cell lines IMR-32 and LA-N-1 [45]. The cell lines, which expressed barely detectable amounts of B2m-free (L31-positive molecules) and B2m-complexed HLA-I (W6.32-and BBM.1-reactive molecules), expressed B2m-free HC monomers upon differentiation with either retinoic acid or serum starvation. The investigators could not alter the percentage of L31-positive monomers by the addition of B2m molecules to the cultures. Based on these findings, Marozzi et al. [46] suggested that the B2m-free HCs are not capable of binding to B2m because of an “altered conformation” of the monomers.

Martayan et al. [47] assessed the role of B2m in regulating the conformation and surface expression of HLA-C molecules on a B2m-deficient kidney carcinoma cell line with the three mAbs L31, W6/32, and Q/128, reactive to alpha 1, 2, and 3 domains of the HCs, respectively. While the carcinoma cells expressed low but significant amounts of free HLA-CW1 HCs at the cell surface, transfection with B2m caused a change in the antibody reactivity of the three domains of HLA-CW1 molecules, thereby providing the first experimental proof to show that assembly with B2m affects the folding of not only the αl and α2, but also of the α3 domain. 

**Figure 1:**
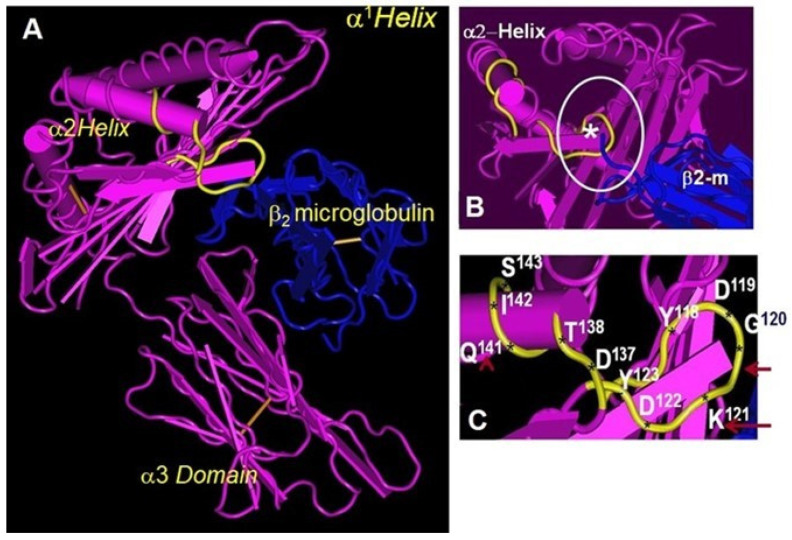
B2m-associated HLA class I heavy chain (pink) showing the conformation of the α1, α2, and α3 domains in the presence of B2m (blue). The orientation of the domains may not be the same in the absence of B2m. (**A**). The amino acid sequences masked by B2m are shown in yellow. In B2m-free HCs, they are exposed and often elicit antibodies in humans, resulting in the production of IgG against the amino acid sequence shared by all HLA class I molecules [41]. The IgG produced against the shared epitope will react with almost all HLA-I antigens coated on the LABScreen beadsets. The LABScreen beadset, in contrast to the Immucor beadset, carries an admixture of B2m-associated and B2m-free HCs [52,53]. (**B**). The shared sequence AYDGKDY (shown within a white circle) is shared by almost all alleles of classical (HLA-A, HLA-B, and HLA-C) and non-classical (HLA-E, HLA-F, and HLA-G) HLA class I isoforms. The sequence AYDGKDY is masked by B2m in intact HLA-I (Face-1) but is exposed in B2m-free HCs (Face-2). The asterisk shows the position of the shared epitope AYDGKDY (yellow) masked by B2m peptide sequence (blue) (**C**). The shared sequence with their position numbers is labelled in white.

We [48,49] have reported that the so-called “HLA-E-specific” mAbs, namely MEM-E/02 [50] and 3D12 [51], bound to several HLA-Ia molecules (HLA-B and HLA-C) coated on microbeads (Table 1), strikingly similar to mAb HC10 [29]. HLA-Ia reactivity of MEM-E/02 and 3D12 was tested in a Luminex flow cytometric single antigen bead assay using LABScreen bead sets. The LABScreen beads, in contrast to Immucore beads, carry an admixture of B2m-associated and B2m-free HCs [52,53]. The binding of these mAbs to B2m-free HLA-E and HLA-Ia molecules was inhibited dosimetrically by the sequence ^115^QFAYDGKDY^123^, which is shared by almost all HLA class Ia and Ib alleles. This sequence is masked by B2m in intact HLA-I (Figure 1A) but exposed in B2m-free HCs. Both MEM-E/02 and other MEM-E series and 3D12 were extensively used to supposedly study HLA-E distribution in human cancer cells (Table 2, [53]). Both MEM-E/02 and 3D12 bound to the sequence ^115^QFAYDGKDY^123^, commonly shared by almost all B2m-free HCs of HLA-I alleles but masked by B2m in native intact B2m-associated HLA-I (Figure 1B) [41,49,54].

Sasaki et al. [55] have further confirmed the relative expression of intact B2m-associated HLA-E and B2m-free HCs of HLA-I during gastric cancer progression using an “HLA-E monospecific mAb” TFL-033 and an “HLA-I polyreactive mAb” MEM-E/02, respectively. The incidence of MEM-E/02 positivity was 40% in adenocarcinoma and 22% in gastric diffuse carcinoma. Using an array of metastatic tumor tissues, we noted that the staining with MEM-2 and TFL-033 is 40% and 20% in peritoneal lesions and 60% and 40% in ovarian carcinoma, respectively. Studying the impact of incubating a peritoneal metastatic cell line with IFN-γ, a marked increase occurred from 40% (0 U/mL) to 100% (200 U/mL) MEM-E/02 positivity; however, for TFL-033, they remained at 0% at concentrations of 200, 400, and 600 U/mL of INF-γ, suggesting the upregulation of B2m-free HCs upon cytokine activation. All the studies above confirm the expression of B2m-free HCs in human cancer tissues, which are probably upregulated by proinflammatory cytokines.

**Table 2:** List of manuscripts where mAbs MEM-E/02 and 3D12 were used for HLA-E detection, assuming that the mAbs are HLA-E specific.

Type of Cancer	HLA-E mAbs *	Citation
Melanoma	MEM-E/02	Derré L et al. 2006 [56]
Lip squamosal cell carcinoma	MEM-E/02	Goncalves et al. 2016 [57]
Laryngeal carcinoma	MEM-E/02	Silva TG et al. 2011 [58]
Vulvar intraepithelial carcinoma	MEM-E/02	van Esch EM et al. 2014 [59]
Penile cancer	MEM-E/02	Djajadiningrat et al. 2015 [60]
Glioblastoma	MEM-E/02	Mittelbronn M. et al. 2007 [61]
Glioblastoma	MEM-E/02	Kren L et al. 2010 [62]
Glioblastoma	MEM-E/02	Kren L et al. 2011 [63]
Oral osteosarcoma	MEM-E/02	Costa Arantes et al. 2017 [64]
Intraoral mucoepidermoid carcinoma	MEM-E/02	Mosconi C et al. 2017 [65]
Rectal cancer	MEM-E/02	Reimers et al. 2014 [66]
Colorectal carcinoma	MEM-E/02	Benevolo M et al. 2011 [67]
Colorectal carcinoma	MEM-E/02	Zeestraten et al.2014 [68]
Colorectal carcinoma	MEM-E/02	Guo et al. 2015 [69]
Colorectal carcinoma	MEM-E/02	Huang et al. 2017 [70]
Gastric cancer	MEM-E/02	Sasaki et al. 2014 [55]
Hepatocellular carcinoma	MEM-E/02	Chen et al. 2011. [71]
Non-small-cell lung carcinoma	MEM-E/02	Talebian-Yazdi et al. 2016 [72]
Breast cancer	MEM-E/02	de Kruijf EM et al. 2010 [73]
Breast cancer	MEM-E/02	da Silva et al. 2012. [74]
Ovarian cancer/cervical cancer	MEM-E/02	Gooden M et al. 2011 [75]
Cervical cancer	MEM-E/02	Gonçalves MA et al. 2008 [76]
Cervical cancer	MEM-E/02	Spaans VM et al. 2012 [77]
Cervical squamous and adenocarcinoma	MEM-E/02	Ferns et al. 2016 [78]
Serous ovarian adenocarcinoma	MEM-E/02	Andersson et al. 2015 [79]
Serous ovarian Adenocarcinoma	MEM-E/02	Zheng et al. 2015 [80]
Renal cell carcinoma	MEM-E/02	Hanak L et al. 2009 [81]
Renal cell carcinoma	MEM-E/02	Kren L et al., 2012 [82]
Thyroid cancer	MEM-E/02	Zanetti et al. 2013 [83]
Hodgkin lymphoma	MEM-E/02	Kren L et al., 2012 [84]
Melanoma, cervical cancer	*3D12*	Marín R et al. 2003 [85]
Glioblastoma stem cells	*3D12*	Wolpert et al. 2012 [86]
Glioblastoma	*3D12*	Wischhusen J et al. 2012 [87]
Neuroblastoma	*3D12*	Morandi et al. 2016 [88]
Hodgkin lymphoma	*MEM-E/02*	Kren L et al., 2012 [84]
Chronic lymphocytic leukemia	*3D12*	McWilliams et al. 2016 [89]
Chronic lymphocytic leukemia	*3D12*	Wagner et al. 2017 [90]
Many cancers	3D12	Sensi M et al.2009 [91]

* These mAbs reacted with B2m-free HCs coated on LABScreen beadsets, and the binding was inhibited by the shared sequences of HLA-I heavy chains (AYDGKDY) [48,49,52,53,54].

Several investigators have discussed the origin of cell-surface B2m-free HCs. Many considered that the B2m-free HC might be formed at the plasma membrane by the dissociation of B2m-associated HCs (for details, see [51]). Capps et al. [92,93] and Lhotakova et al. [94] have shown that B2m-free HCs can also exist in the ER and Golgi complex of B2m-defective, virus-infected, and tumorigenic cells, from which they can travel through the secretory pathway to reach the cell surface. These findings led them to hypothesize the presence of two different kinds of B2m-free HCs (Face-2), one with a propensity to rebind to B2m [95] and another incapable of re-associating with B2m and peptide but remaining as Face-2 due to a conformational change in the HC [45,96,97]. Indeed, the open nature of Face-2 molecules promotes binding between two Face-2 molecules, leading to the formation of homodimers (Face-3) and heterodimers (Face-4).

## 8. Formation of Cell-Surface B2m-Free Face-3 (Homodimers) by Face-2 (HC Monomers) in Humans and Mice

In HLA-B27 transgenic mice that developed arthritis in the absence of B2m and MHC class II, Allen et al. [98] showed that the B2m-free HCs can form disulfide-bonded homodimers, depending on the Cys^27^ residue in their extracellular domain. Interestingly, they noted that despite the absence of B2m, the dimerized B2m-free HCs are stabilized by a peptide epitope. In addition, mAb W6/32 recognized these dimers, suggesting that mAb W6/32 can bind to S-S bonded HCs. They also pointed out that the B-27 homodimers can induce spondyloarthropathy in B2m-deficient mice.

Boyson et al. [39] have also documented the presence of HLA-G HC homodimers, using mAb MEM-G/1 generated against denatured HLA-G HC. They have also generated mAb MEM-G/11 against B2m-associated HC with a peptide. Interestingly, the mAb MEM-G/1 revealed specificity for extravillous trophoblast. MEM-G1-stained electropherograms of the cell lysates purified by gel filtration confirmed the presence of monomers (33 kDa) and dimers (66 kDa). The dimer was reduced to monomers under reducing conditions, indicating that the dimers were S-S bonded, and the HLA-G HC is capable of forming dimers under natural conditions. Moreover, the amino acid sequence of HLA-G HC revealed the presence of two free cysteines, Cys-42 and Cys-147, not commonly found in all HLA class I molecules. The Cys-42 resided on a loop between the third and fourth beta strands of the alpha 1 domain. Cys-147, in contrast, was located on the alpha helix. Because of its accessibility, Cys-42 is considered to be the most likely participant in an intermolecular disulfide linkage. Gonen-Gross et al. [99,100] further confirmed the homodimerization of HLA-G HCs and formation of s-s bonds between the Cys^42^ of two HC monomers and between a Cys^42^ of the alpha 1 domain and a Cys^147^ of the alpha 2 domain of two different HLA-G HCs.

Several reports suggest that Face-2 dimerization is promoted by the clustering of Face-2 on the cell surface. Chakrabarti et al. [95] observed “self-association” of fluorescing Face-2 of HLA-A2 HCs in liposomes and on the cell surface membranes due to clustering. The molecular proximity of the self-associated Face-2 molecules was determined by flow cytometric phosphorescence resonance energy transfer (FCET). They visualized the aggregation of the HCs by monitoring fluorescence photo-bleaching recovery (FPR) and time-resolved phosphorescence anisotropy (TPA) in addition to FCET. The addition of B2m to the liposomes blocked the self-association. HLA aggregates were also observed on the surface of human lymphoblastoid (JY) cells. 

Similarly, Matko et al. [96] detected the clustering of Face-2 molecules on activated normal B and T cells, on cells of B and T lymphoblast lines, and on transformed fibroblasts. The Face-2 clustering correlated with the presence of the HC10 epitope of Face-2 at the cell surface. However, no such clustering was observed on the surfaces of resting B or T cells or normal fibroblasts.

Damjanovich et al. [101] also confirmed the occurrence of HLA Face-2 clusters on the plasma membranes of human T (HUT-102B2) and B (JY) lymphoma cells. The possible role of Face-2 oligomerization (homo- and heterodimerization of HCs) was further validated on the B lymphoblastoid cell line JY [102]. Khare et al. [29] demonstrated that the HLA HC homo- and heterodimer variants can function like HLA class II molecules. Using super-resolution microscopy, Kennedy et al. [103] noted that the cell surface clustering of HLA-I alleles (HLA-B*27:05/B*53:01/B*57:01 and HLA-C*06:02/C*07:02) transfected into LCL 721.221 lymphocytes that lacked the expression of classical HLA-I differed among different alleles. The density of clustering of HLA-C on the cell surface was greater than that for HLA-B, revealing that different loci exhibit distinct nanoscale organizations at the cell surface. 

Capps et al. [92] and Capps and Zuniga [93] documented in vivo dimeric association between HLA-I HCs in a murine model system using antiserum raised against an amino acid sequence of the HCs of H-2L^d^ and H-2D^b^. The immunoblots of cell lysates revealed the presence of both homodimers (Face-3) and heterodimers (Face-4) of B2m-free HCs. Allen et al. [98] showed homodimerization of Face-2 on the cell surface of HLA-B27-transfected T2 cells. The homodimerization is facilitated by disulfide bonding between the Cys^67^ of two Face-2 HCs. Interestingly, the HLA-B27 Face-3 homodimers are recognized by both Face-2 binding mAb HC10 and Face-1 binding mAb W6/32. In this regard, Mear et al. [104] pointed out that the homodimerization results from HLA-B27 misfolding within the ER, the accumulation of which occurs concomitantly with the proinflammatory intracellular stress response. Such misfolding of HLA-B27 may contribute to its recognition by both HC10 and W6/32. However, Bird et al. [105] documented that HLA-B27 homodimers form in the ER but appear unable to egress to the cell surface in human cells. Cell surface HLA-B27 homodimers are abundantly expressed in a variety of lymphoid cell lines. They have shown with inhibition experiments that HLA-B27 homodimers can arise from cell-surface heterodimers via an endosome-dependent recycling pathway. Tran et al. [106] found a correlation between disease incidence and HLA-B27-dimers in the HLA-B27 transgenic rat models. 

## 9. B2m-Free Face-3 (Homodimers) and Face-4 (Heterodimers) on Exosomes and on the Cell Surface

Exosomes are 50–150 nm vesicles formed by the inward budding of endosomes to generate multivesicular bodies. A fraction of these can fuse with the cell surface plasma membrane and release their internal vesicles to the extracellular environment. Epithelial cells [107], reticulocytes [108], neurons [109], mast cells [110], T and B lymphocytes [111], dendritic cells [112,113,114,115], and even cancer cells [116,117] generate exosomes. Lynch et al. [118] have shown that exosomes from the Jesthom cell line (the human EBV-transformed B cell line) form dimers between HLA-A and -B molecules (though at lower levels due to steric constraints caused by the relative positions of the cysteines in the cytoplasmic tails). These dimers can be detected after the release of exosomes from human monocyte-derived dendritic cells. Similarly, Makhadiyeva et al. [119] showed that similar Face-3 dimers are present on the human lymphoblastoid lines LCL.221, CEM cell lines, Jesthom cell line, and on the rat C58 lymphoma cell lines when the redox environment has been significantly altered, either by chemical oxidation with diamide, chemically induced apoptosis with hydrogen peroxide and thimerosal, or by the cross-linking of FacR/CD95. Interestingly, in the CEM cell lines, there were low levels of HLA-B27 dimers in the cell lysates in the absence of oxidative stress. In contrast, the Jesthom cell line, which expresses a higher level of HLA-B27 than the CEM cell lines, displayed dimers under normal conditions. Santos et al. [38], while determining to what extent the cell activation could induce HLA-B27 dimers in human monocyte-derived dendritic cells, noted very low quantities of dimers in HLA-B27-negative dendritic cell cultures and indicated that other non-HLA-B27 HCs may form Face-3 and Face-4 dimers. Morales et al. [120] investigated the ability of primary human placental villous cytotrophoblast cells (vCTB cells) from term placentas to synthesize monomeric and dimeric forms of HLA-G5 and tested disulfide bonding. The vCTB cells produced dimers migrating to a molecular weight (MW) of ~74 (kDa) under nonreducing conditions. Under reducing conditions, the dimers readily dissociated to yield monomers at ~37 (kDa) MW. They further indicated that the two extra cysteine residues that characterize HLA-G, Cys^42^ and Cys^147^, are associated with disulfide-linked oligomerization. Because there are no cysteines within the 21 amino acids encoded by HLA-G5 intron 4, it is inferred that Cys^42^ and Cys^147^ form the disulfide bonds. Most importantly, their experiments showed that vCTB cells synthesize B2m mRNA at comparatively low but readily detectable levels but do not demonstrate any immunohistochemically detectable B2m protein associated with their HLA-G5 H-chains.

## 10. Folding and Conformational Orientation of HLA-I HCs with or without B2m

Martayan et al. [47] specifically questioned the differences in the folding and conformational orientations between B2m-associated (B2m transfectant cells) and B2m-free HLA HCs (B2m-deficient cells) in a kidney carcinoma cell line (KJ29 cells) that is positive for HLA-A2, -B27, and –Cw1 and does express low but significant amounts of free HLA-Cw1 HCs at the cell surface in B2m-deficient cells. To assess the role of B2m in regulating the conformation and surface expression of HLA-C molecules, three different mAbs binding to the alpha 1 domain (mAb L31), alpha 2 domain (W6/32), and alpha 3 domain (Q1/28) of HLA-I were employed. Antibody binding and phase partitioning experiments revealed that free HLA-CWl HCs are more unfolded than free HLA-A2 and -B27 HCs. By comparing the mAb Q1/28 binding to the α3 domain of HLA-CWl HCs with those of HLA-A2 and -B27 HCs before and after their assembly with B2m, it was noted that the Q1/28 epitope may be masked in CW1 HCs due to interactions with other HCs, indicative of homo- or even heterodimeric assembly. These observations suggest that all three alpha domains may be exposed in free HCs, whereas the HCs associated with B2m or other monomers may mask the α3 domain. In dimers, the α1 and α2 domains are exposed for cysteine-cysteine interaction or tyrosine-tyrosine interaction due to the presence of a heavy load of tyrosine (see Table 3 in Ref. [4]).

## 11. Do Conformational Alterations of the Cell Surface B2m-Free Human HLA HCs (Proto-HLA: Faces-2, -3, and -4) Enable Novel Immunomodulating Functions?

B2m-associated HLAs (Face-1) provide a stable groove for peptide binding and enable the presentation of the peptide to the CD8+ T lymphocytes. In addition, there are reports suggesting the interaction between Face-1 and epidermal growth factor receptors [121], insulin [122], insulin receptors [123], and gamma-endorphin [124]. As noted above, several investigators have recognized conformational alterations in the B2m-free monomeric as well as in dimeric HCs. Possibly in the absence of B2m, the HCs may not have the grooves to hold peptides for antigen presentation. Supportingly, Ortis-Navarrete and Hammerling [125] and Carreno and Hansen [126] have observed that at the cell surface, B2m-free HCs (Face-2 molecules) are incapable of regaining a native alpha 1 and alpha 2 domain conformation upon peptide addition. However, reports presented in the next section not only document that the alpha 1 and alpha 2 domains of B2m-free HCs are capable of binding with a variety of polypeptides and proteins, but also elucidate novel functions of conformationally altered B2m-free monomeric and dimeric HCs, in association with receptors on natural killer cells and lymphocytes. 

## 12. The α1 and α2 Domains of B2m-Free HCs as Ligands for KIR and LIR

**Figure 2:**
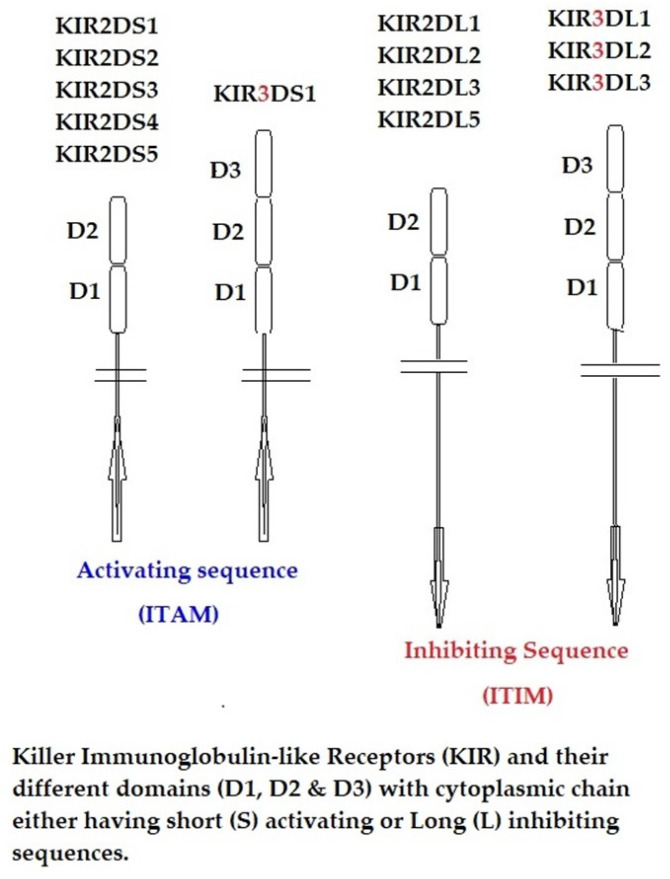
Protein structures of the members of immunoregulatory killer-cell immunoglobulin-like receptors (KIRs). Each receptor may have two or three protein domains with a short or long cytoplasmic chain. The KIR inhibitory receptor long (L) chains are associated with immunoreceptor tyrosine-based inhibitory motif (ITIM), and the activating receptor short (S) chains are associated with immunoreceptor tyrosine-based activating motif (ITAM). The ligand–receptor interaction of the signaling takes place through the charged amino acid in the terminal of the KIR transmembrane domain.

Natural killer (NK) cells, formed in the bone marrow, are components of innate immunity responsible for killing virus-infected and cancer cells. Their cell surface markers include CD56 and CD16, and they also possess a family of genes on chromosome 19q13.4 in the “leukocyte receptor complex” for immunoglobulin-like glycosylated cell surface monomeric proteins, which include killer immunoglobulin-like receptors or KIRs. KIRs possess two to three extracellular Ig domains, designated as KIR2D and KIR3D, that include both inhibitory and activating forms. Activating KIRs have a short cytoplasmic tail (KIR2DS and KIR3DS) containing immunoreceptor activating motifs that interact with HLA (Face-1)-activating ligands. The inhibitory KIRs have a longer cytoplasmic tail (KIR2DL and KIR3DL) containing immunoreceptor-tyrosine-based inhibitory motifs (ITIM) [127,128,129,130]. Debska-Zielkowska et al. [130] recently provided nomenclature based on the long or short cytoplasmic tails; e.g., KIR3DL1 represents an inhibitory receptor with three Ig domains with a tail possessing ITIM, whereas KIR3DS1 is non-inhibitory with a short cytoplasmic tail without ITIM but with activating motifs (also referred to as ITAM). The Ig domains are numbered 2 and 3, as in KIR2D and KIR3D. Figure 2 illustrates KIR protein structures showing different domains and cytoplasmic chains and their activating and inhibiting sequences. Most of these KIR members share a sequence identity greater than 90%. KIR-like receptors are also expressed by a small subpopulation of CD8+ αβ T cells and γδ T cells [131,132]. Similar to the KIRs of NK cells, leukocytes also express immunoglobulin-like receptors (referred to as LILRs). LILRs are expressed on B cells, T cells, and monocytes/macrophages (as LILRB1) and on dendritic cells, monocytes, and macrophages (as LILRB2) [133,134]. The LILR family includes both inhibitory receptors (LILRB) on human lymphoid and myelomonocytic cells and activating receptor members (LILRA). The cytoplasmic tail of both LILRB1 and LILRB2 receptors incorporate immunoreceptor tyrosine-based inhibitory motifs (ITIMs), which are phosphorylated upon cell activation and inhibit leukocyte activation through SHP phosphatase recruitment.

Early studies documented interactions between different KIRs with B2m-associated HLA class I molecules. It was also reported that KIRs bind to α1 and α2 domains of B2m-associated HLA; furthermore, the associated peptides are considered crucial for KIR recognition [135,136,137]. Peptides associated with B2m-associated HLA generate stable and proper folding of the HC of the molecules at the cell surface. Conformational orientation of the α1 and α2 domains of the HCs differ markedly in the presence of both a peptide and B2m, and similarly, the binding affinity of KIR receptors to α1 or α2 domains also differs. During the periods of investigation, many were not aware of the existence of B2m-free HCs and, above all, the possibility of KIR association with altered conformations of α2 or α1 domains of the HCs. 

In subsequent years, reports began to emerge documenting that in addition to B2m-associated HLA, B2m-free HLA monomers and homodimers can also serve as ligands for KIRs and LILRs. The credit for this unique discovery goes to Simon Kollnberger and his team, who have extensively contributed to our understanding of the interaction of KIRs and LILRs with B2m-free HLA HC monomers and homodimers. Kollnberger et al. [138] have shown that patients with spondylarthritis express mAb HC10-reactive cell-surface B2m-free HLA-B27 HC homodimers (referred to as HC-B27) on monocytes and lymphocytes ex vivo as well as on the peripheral blood monocytes of the patients, strongly recalling early reports that showed activated human lymphocytes express conformationally distinct B2m-free HCs [22] that cluster on the cell surface [60]. Kollnberger et al. [138] used fluorescent tetrameric complexes of HC-B27 to document the binding of the cell-surface HC-B27 (B2m-free HC of B27) and their homodimers and tetramers with KIR3DL1, KIR3DL2, and LILRB2 receptors. They [139] report that KIR3DL1 recognition of B2m-free HCs is dependent on residues 77–83 of the class I α helix. Furthermore, they consistently document, in repeated experiments, the binding of HC-B27 to KIR3DL2. They suggest that a conformational change upon HC-B27 dimerization permits binding to KIR3DL2. They [102] further document that HC-B27 binding to T cells correlated with KIR expression, and that mAb against KIR3DL1 and KIR3DL2 blocked HC-B27 staining of a KIR3DL1-expressing NK line. Furthermore, it was documented that HC-B27 homodimers bind to ILT4 (LILRB2), an inhibitory receptor expressed on monocytes, macrophages, and dendritic cells.

Kollnberger et al. [139] compared the role of peptides, which are complexed with B2m-associated HLA-B27, on the interaction of B2m-associated HLA-B27 and B2m-free HC27 homodimers with KIR3DL1 and KIR3DL2 as well as with LILRB1 and LILRB2. The peptides used include three naturally processed self-peptides and three pathogen-derived epitopes bound to KIR3DL1-expressing transfectants and NK cells. The HC-B27, but not the B2m-free HCs of HLA-A2, B7, or B57, formed homodimers in the presence of the peptide epitopes. HC-B27 bound to KIR3DL1, KIR3DL2, and LILRB2 but not LILRB1. However, in contrast to B2m-associated HLA-B27, the HC-B27 homodimer binding to KIR did not depend on the peptide. On the other hand, both B2m-associated HLA-B27 and HC-B27 homodimer binding to LILBR2 remained independent of the peptides or their sequences. Strikingly, KIR3DL2 ligation by B27 HCs inhibited NK and T cell IFN-γ production. The KIR3DL2 ligands also included HLA-A3 and HLA-A11 [140]. 

Like human KIRs and LIRs, mice (and rats) express paired Ig-like receptors, designated as PIRs, which include both activating PIR-A and inhibitory PIR-B receptors, on B cells and monocyte/macrophage lineage cells. Kollnberger et al. [141] immunoprecipitated murine PIR-A and -B from the RAW264.7 macrophage cell line, and murine PIR-A from the J774.A1 line, using homodimers of HC-B27. The binding of homodimers of HC-B27 to PIR is inhibited by HC10, an HLA-HC-binding mAb, and the mAb that ameliorates arthritis in HLA-B27-B2m−/− mice. These findings indicate that the interaction of HC-B27 homodimers with immune receptors on cells of the myelomonocytic lineage or on B lymphocytes might be involved in the pathogenesis of spondyloarthritis.

Kollnberger and Bowness [142] hypothesize that infection may stimulate the production of cell-surface B27 dimers by antigen-presenting cells, either by direct mobilization from an intracellular pool or, more probably, indirectly, from recycling unstable B2m-associated B27 heterotrimers. They [143] further speculate that the binding of the HC-B27 homodimer to KIR and LILR receptors could then promote inflammation by enhancing the survival of proinflammatory KIR-expressing NK and T cells and influence the differentiation of LILR-expressing antigen-presenting cells.

In subsequent studies, Kollnberger’s team [32,143] have conclusively shown that B27 monomers (HC-B27) and homodimers (HC-B27_2_) bind to LILRB2 and KIR3DL2 with a stronger avidity than the intact B2m-associated HLA-B27. LILRB2 bound to B27 HCs and dimers expressed by transfected cells. HC-B27 homodimer-expressing antigen-presenting cells (APCs) inhibited the production of IL-2 by LILRB2-transduced Jurkat T cells more strongly than APCs expressing other HLA class I molecules. They concluded that the stronger binding of HC-B27 monomers and homodimers to LILRB2 could play a role in the pathogenesis of ankylosing spondylitis. They also showed that the binding of B27 monomers (HC-B27) and homodimers (HC-B27_2_) stimulated greater KIR3DL2 phosphorylation than HLA-A3 and IL-2 production by T cells.

Kollnberger’s team [144] further compared the affinity of KIR and LILR to B2m-associated and B2m-free HC monomers and dimers of HLA-B*27:05, which is associated with ankylosing spondylitis (AS), and HLA-B*27:09, which is not associated with AS. HLA-B*27:09 formed fewer B2m-free HCs and homodimers than HLA-B*27:05. HLA-B*27:05-expressing cells stimulated KIR3DL2-CD3 reporter T cells more effectively, and the cells expressing HLA-B*27:05 promoted the survival of KIR3DL2-positive NK cells more strongly than those expressing HLA-B*27:09. Increased proportions of NK and CD4 T cells expressed KIR3DL2 in HLA-B*27:05+ AS patients compared with healthy controls, who are HLA-B*27:05+, HLA-B*27:09+, and HLAB27-.

Based on these findings, Kollnberger’s team [145] visualized the immune-pathological events taking place after the expression of cell-surface B2m-free human HLA-I (Proto-HLA) and mouse H-2 mono- and dimeric molecules, as follows:

- B2m-free B27 HCs (HC-B27) are first induced by bacterial infection.
- Subsequently, KIR3DL2 binding to HC-B27 affects the polarization of IL-17-producing immune cells by inhibiting the IL-2 and IFN-γ brakes on IL-17 production.
- Stronger binding of HC-B27 to KIR3DL2 and LILR compared with other B2m-associated HCs of HLA is capable of promoting the differentiation of pathogenic T and innate lymphocyte cell subsets, initially at mucosal sites.
- Subsequently, these cells migrate to affected joints, where they could amplify inflammation by activating fibroblast-like synoviocytes (FLSs) and antigen-presenting cells (APCs), releasing inflammatory mediators that damage joints.
- B27–KIR3DL2 interactions between FLSs and/or APCs and leukocytes promote the expansion and differentiation of these cells and recruit other immune cells to inflamed joints.
- Differentiated T and innate lymphocyte cells amplify immune responses by promoting antigen-presenting cell polarization [146].
- HC-B27-KIR3DL2 interactions could promote the differentiation of IL-17-producing immune cells by reducing T cell signaling strength and/or by inhibiting immune cell production of IFN-γ.
- HC-B27 could promote the differentiation of IL-17-producing immune cells by influencing immune synapse formation.
- HC-B27-LILRB2 or other LILR interactions could amplify immune responses by inhibiting the formation of regulatory T cells and tolerogenic dendritic cells.
- Both Th17 cells and NK cell subsets stimulate bone turnover by stimulating osteoclasts [147].

While the LILRA1; A4, A5, and A6; and LILRB2 molecules are expressed by cells of the myelomonocytic lineage [148], LILRB2 is expressed by human hematopoietic stem cells [149], and LILRB5 transcripts are detected in natural killer (NK) cells [150], mast cells [151], macrophages, and osteoclasts [115]. Tedla et al. [151] reported the intracellular expression of LILRB5 in mature human mast cells. Previously, Kollnberger et al. [139] and Giles et al. [143] showed that B2m-free HCs of HLA-B27 (HC-B27) bind to LILRA1 and LILRB2 receptors. Kollnberger’s team [152] identified LILRB5 as a new receptor for B2m-free HCs of HLA. LIRB5 bound specifically to HC-B27 homodimers but did not bind to B2m or peptide-associated HLA-A3, HLA-B7, and HLA-B27 heterodimers. Surface expression of LILRB5 was primarily detected on peripheral monocyte cells. LILRB5 but not LILRA5 co-immunoprecipitated with HC-B27 in transduced cells.

These studies clearly document that conformational change in the α1 and α2 domains of HLA HCs in the absence of B2m and a peptide can still be recognized strongly by KIR and LILR receptors facilitating immunomodulation. These findings open new avenues to immunotherapy of autoimmune diseases and even human cancers that express B2m-free HCs even upon depletion of cell surface intact HLA class I molecules.

## 13. Conclusions: Evolutionary Implications of B2m-Free HCs

This review examined evidence supporting the existence of B2m-free HCs on the cell surface of human and murine cells in normal and pathological conditions. While early investigators believed that the cell surface HLA HCs are stable only when associated with B2m [153,154]. The stable expression of B2m-free HCs was well documented well on the cell surface of B2m-deficient mice [155] and humans [22,23,24]. It was suggested that the B2m-free HCs were formed due to the dissociation of B2m from the B2m-associated heterodimer [22,23,24,25], but findings on spondyloarthritides revealed that stable B27-HC can exist without B2m. Findings made on human cancers [53,55] also revealed that cancer cells of different organs consistently express B2m-free HCs.

Interestingly, Bix and Raulet [156] documented that the peptides may be able to bind free HCs with sufficient affinity to establish functionally conformed molecules on the cell surface. Recognition of stable B2m-free HCs on the cell surface led to the discovery of their dimers upon clustering both on exosomes [95] and on the cell surface [96,97]. The dimerized B2m-free HCs are further stabilized by peptide binding [97]. These findings led to the concept that B2m association with the HC could be a later event that may have occurred during or after homo- and heterodimerization of HC monomers. Indeed, HLA class II is a typical example of heterodimerization (Face-4) from monomeric HCs (Face-2). Both homodimerization and heterodimerization of HCs with or without B2m generate conformational grooves to hold a peptide for presentation as antigens to adaptive immune cells. Therefore, it appears that the existence of monomeric HCs and their dimerization during the clustering of cell surface monomers seems to be a phylogenetically conserved event [4]. The structural patterns of HCs and the heterodimerization of MHC class I in Chondrichthyes (sharks), Teleostei, and tetrapod species support the phylogenetic concept [157,158,159,160,161]. Indeed, Dijkstra et al. [157,158,159,160,161] proposed that the structural patterns of HCs are evolutionarily primitive among HLA-I and HLA-II classes; Kaufman et al. [162,163,164] postulated that B2m-free HC homodimers would have evolved into class II heterodimers; Wu et al. [154] hypothesized that in evolution, a class II-like molecule would have come first, after examining the detailed structure of the HCs of HLA-I and HLA-II. Strikingly, Hashimoto et al. [165], based on sequence similarities, proposed that HLA class I and class II molecules were most likely evolved from a common ancestor. They report that the class II b-chain may have diverged more slowly than other chains more than 400 million years ago, and the HLA HCs had membrane-proximal domains of the same length as the contemporary HCs of class II.

In a recent study [4], examining the amino acid sequences of HC of HLA-A, HLA-B, HLA-C, HLA-E, HLA-F, and HLA-G, we showed that several amino acid sequences are shared among them, and most particularly, ^117^AYDGKDY^123^ is shared by almost all. Similarly, we observed that a six-amino-acid sequence ^34^VRFDSD^39^ with a neighboring double RA and a neighboring triple ^59^EYW^60^, found in all classical HLA class I isomers, are also in the B chain of HLA class II isomers, DRB, DQB, and DPB. These findings strengthen our proposal hypothesizing that B2m-free HCs may indeed represent a “Proto-HLA” and the common progenitors of the HLA-I and HLA-II classes [4].

If this is further validated, it would imply that the conformational exposure of the α1 and α2 domains of HCs would serve as ligands for immune cell receptors (such as KIR and LILR) even early during vertebrate evolution. It appears that the interaction of the B2m-free HCs with KIR and LILR may represent early receptor–ligand interaction between cells of both innate (e.g., NK) and adaptive (e.g., T and B lymphocytes) immune systems, even before the monomeric HCs and dimeric HCs are stabilized with an additional peptide. Considering the phylogenetic primitiveness of HCs (as discussed in [4]), these functions may be early immune functions of these B2m-free HLA HCs or Proto-HLAs.

More studies are needed to document the ability of B2m-free HC monomers, homodimers, and heterodimers to present peptides to receptors of the adaptive immune systems in fishes, amphibians, reptiles, and birds. Furthermore, the evolution of the association of such HCs with B2m needs to be better clarified to gain insight into the mechanics of adaptive immunity. Finally, we hypothesize that both the binding of B2m-free HCs on the surface of cancer cells to KIRs with long (L) inhibiting sequences (ITIM) may lead to cancer immune escape. The binding of B2m-free HCs formed during inflammation of organ transplants to KIRs with short (S) activating sequences (ITAM) may lead to transplant rejection. Although more investigations are needed, targeting shared epitopes such as AYDGKDY exposed on the B2m-free HCs, using mAbs such as TFL-006 [166,167] on human cancers and inflamed organ transplants, could open up a promising new avenue of antibody-based passive immunotherapy.

## Figures and Tables

**Table 1:** The commercial mAbs claimed to be specific for HLA-E, namely MEM-E/02 [49,50] and 3D12 [51], tested at 1/10 dilution, reacted with B2m-free HCs of almost all HLA-I antigens coated on the LABScreen beadsets [47,48]. The LABScreen beads carry both B2m-associated as well as B2m-free HCs [52,53]. The values are expressed as mean fluorescence intensity (MFI). Blank regions refer to MFI below 500 (0–499), which is considered to be negative.

HLA-Ia alleles	mAb MEM-E/02	mAb 3D12	HLA-Ia alleles	mAb MEM-E/02	mAb 3D12	HLA-Ia alleles	mAb MEM-E/02	mAb 3D12
A*01:01			B*07:02	1910		B*52:01	928	1416
A*03:01		789	B*08:01	1062		B*53:01	2754	
A*11:01	2940		B*13:01	5700	1820	B*54:01	1910	
A*11:02	559		B*13:02	1326	1171	B*55:01	1287	
A*23:01	4096		B*14:01	3135		B*56:01	5352	
A*24:02	2505		B*14:02	942		B*57:01	3626	588
A*24:03	629		B*15:01	832		B*57:03	2586	1143
A*25:01	1593		B*15:02	3250		B*58:01	1636	823
A*29:02	526		B*15:03	4731		B*59:01	2803	
A*30:01			B*15:10	768		B*67:01	704	1856
A*30:02	603		B*15:12	1903		B*73:01	5560	659
A*32:01	3037		B*15:13	3400	591	B*78:01	4273	
A*33:01	1604		B*15:16			B*81:01	1097	579
A*33:03	991		B*18:01	4392		B*82:01	5295	
A*34:01	1219		B*27:05	942				
A*36:01	571		B*27:08	1175	3264			
A*66:01	664		B*35:01	8716	566			
A*68:01	917		B*37:01	3444				
A*69:01	3125		B*38:01	968	1672			
C*01:02	2567	966	B*39:01	3010				
C*02:02	1713	720	B*40:01	3478	800			
C*03:02	2358		B*40:02	2442	712			
C*0303	2585	571	B*40:06	9898	3216			
C*03:04	1765		B*41:01	4987				
C*04:03	9263	3796	B*42:01					
C*05:01	3076	931	B*44:02	2621				
C*06:02	6680		B*44:03	2654	1321			
C*07:02	2481	1640	B*45:01	3134	604			
C*08:01	1692	592	B*46:01	3042				
C*12:03	1889	1020	B*47:01	777				
C*14:02	2688		B*48:01	3577				
C*15:02	1128		B*49:01	1588				
C*16:01	1869	530	B*50:01	769				
C*17:01	7779	5554	B*51:01	2485	841			
C*18:02	4096	1095	B*51:02	2303				

## Abbreviations

B2m: beta2-microglobulin; BFA: brefeldin A; CTL: cytotoxic T lymphocytes; Cys: cysteine; Face-1: Intact B2m-associated HLA HC; Face-2: B2m-free HC; Face-3: homodimers of B2m-free HCs; Face-4: heterodimers of B2m-free HCs; HCs: heavy chains; IFN: interferon; KIR: killer cell receptor; mAbs: monoclonal antibodies.
